# Selenium—More than Just a Fortuitous Sulfur Substitute in Redox Biology

**DOI:** 10.3390/molecules29010120

**Published:** 2023-12-24

**Authors:** Luisa B. Maia, Biplab K. Maiti, Isabel Moura, José J. G. Moura

**Affiliations:** 1 LAQV, REQUIMTE, Department of Chemistry, NOVA School of Science and Technology | NOVA FCT, 2829-516 Caparica, Portugal; isabelmoura@fct.unl.pt (I.M.); jose.moura@fct.unl.pt (J.J.G.M.); 2 Department of Chemistry, School of Sciences, Cluster University of Jammu, Canal Road, Jammu 180001, India

**Keywords:** selenium in biology, selenoproteins, formate dehydrogenases, hydrogenases, glutathione peroxidases, thioredoxin reductases, iodothyronine deiodinases, human health

## Abstract

Living organisms use selenium mainly in the form of selenocysteine in the active site of oxidoreductases. Here, selenium’s unique chemistry is believed to modulate the reaction mechanism and enhance the catalytic efficiency of specific enzymes in ways not achievable with a sulfur-containing cysteine. However, despite the fact that selenium/sulfur have different physicochemical properties, several selenoproteins have fully functional cysteine-containing homologues and some organisms do not use selenocysteine at all. In this review, selected selenocysteine-containing proteins will be discussed to showcase both situations: (i) selenium as an obligatory element for the protein’s physiological function, and (ii) selenium presenting no clear advantage over sulfur (functional proteins with either selenium or sulfur). Selenium’s physiological roles in antioxidant defence (to maintain cellular redox status/hinder oxidative stress), hormone metabolism, DNA synthesis, and repair (maintain genetic stability) will be also highlighted, as well as selenium’s role in human health. Formate dehydrogenases, hydrogenases, glutathione peroxidases, thioredoxin reductases, and iodothyronine deiodinases will be herein featured.

## 1. Introduction

Discovered in 1817, selenium was for long regarded as a toxic element [1,2,3] and only in the second half of the XX century was it demonstrated to be essential for all forms of life [4,5,6,7,8,9,10]. Living organisms have learned to harness the unique chemical features provided by selenium (over sulfur) and use this element mainly in the active site of oxidoreductases in the form of selenocysteine, an amino acid genetically encoded by a specific codon (UGA) that is considered the 21st amino acid.

Several selenocysteine-containing enzymes evolved to play essential roles in various biological processes. Still, some of those selenoproteins have fully functional cysteine-containing homologues and some organisms do not use selenocysteine at all. Hence, understanding the biological use of selenium is of considerable interest.

Herein, selected selenocysteine-containing enzymes will be described to highlight the biological versatility afforded by selenium, emphasizing the unique chemical features introduced by this element but also drawing attention to interesting cases where both selenium (selenocysteine) and sulfur (cysteine) are known to be catalytically competent. After briefly highlighting the chemical differences between selenium and sulfur (Section 2), formate dehydrogenase (FDH) (Section 3), one of the first enzymes demonstrated to contain selenium, will be discussed in a deeper detail, followed by hydrogenases (Hase) (Section 4). Concise accounts on glutathione peroxidases (GPx) (Section 5), thioredoxin reductases (TrxR) (Section 6), and iodothyronine deiodinases (Dios) (Section 7) will follow. A review of the relevance of selenium for human health will also be included (Section 8).

## 2. Selenium versus Sulfur

Selenium is a chemical element belonging to the chalcogens family of the Periodic Table (Group 16). It resembles the “lighter” sulfur in some chemical features and, in Biology, selenium can be found replacing sulfur in two amino acids: selenocysteine (Se-Cys) and selenomethionine (Se-Met). However, in spite of the similarities, many significant chemical differences exist between these two chalcogens [11,12,13,14,15]. As sulfur, selenium can display a wide range of oxidation states (from −2 to +6), but its preference for lower oxidation states and higher reactivity sets it apart from sulfur. Its reactions are often also faster than its sulfur counterparts because selenium is more polarizable (softer). Its larger spin—orbit coupling (compared to sulfur) probably facilitates spin-forbidden reactions, as the ones involved in the rapid oxidation of selenocysteine under air (compared to cysteine oxidation). Moreover, the selenocysteine selenol’s lower p*K*_a_ value (5.2, compared to 8.3 of cysteine thiol) is expected to favor its deprotonation and nucleophilic character at physiological pH [16], while the weaker Se-H bond makes the selenocysteine less basic, compared to cysteine [17,18]. The biologically relevant redox chemistry is also significantly different in these two elements [19,20,21]. The selenocysteine one-electron oxidation-derived radical is more easily formed ((RSe^•^/RSeH) = 0.43 V versus (RS^•^/RSH) = 0.92 V [22]) and relatively more stable than the cisteine radical [22,23,24]. As a result, for example, while the cysteine radical can oxidize a tyrosine residue (to yield tyrosine radical), the selenocysteine radical can not [22]. Also noteworthy are the thiol/disulfide exchange reactions, where the selenocysteine reactions (Se-Cys/Cys-Se-Se-Cys) are faster than the cysteine ones [12,14,25,26].

This different chemistry suggests that the incorporation of a selenocysteine or a cysteine should modulate the enzyme catalytic activity, with a selenocysteine being able to perform roles that a “common” sulfur-containing cysteine can not. As such, selenium should not be just a fortuitous sulfur substitute in Biology. However, as will be discussed below, there are striking examples where the replacement of selenocysteine by cysteine does not affect the outcome of the biological reaction.

## 3. Formate Dehydrogenase

FDH was one of the first enzymes demonstrated to contain selenium and a selenocysteine-specific codon (TGA) in its gene sequence (*Clostridium thermoaceticum* and *E. coli* enzymes) [27,28]. Those seminal works were essential to overcome the prevailing idea that selenium was (only) a toxic substance and lead to its recognition as an essential element (also for mammals and humans by contemporary works).

In spite of being one of the most widely distributed selenoproteins (probably due to its extensive lateral gene transfer, together with the corresponding selenocysteine synthesis and incorporation system) [29], FDH constitutes a key example where, as far as is presently known, selenium does not present any clear advantage over sulfur. Contrary to other selenoenzymes, living organisms hold both active selenocysteine- and cysteine-containing FDH homologues and, thus, the selenium role in FDH catalysis remains, so far, elusive.

### 3.1. The Current Picture

#### 3.1.1. Enzymatic Machinery

FDHs catalyze the two-electron interconversion of formate and carbon dioxide (Equation (1)) in diverse metabolic pathways, operating in different subcellular locations, such as C1 metabolism, carbon dioxide fixation (carbon assimilation), and to derive energy (coupling formate oxidation to the reduction of different terminal electron acceptors) [30,31,32,33,34,35,36,37,38]. Since each pathway requires a specific “FDH enzymatic machinery” to accomplish the respective biological function, FDHs evolved as a highly heterogeneous group of enzymes, displaying diverse structural (subunits) organization and composition of redox-active centers (Table 1) [39,40,41,42,43,44,45,46,47,48].

HCOO^−^ ⇌ CO_2_ + 2e^−^ + H^+^ (1)

**Figure 1:**
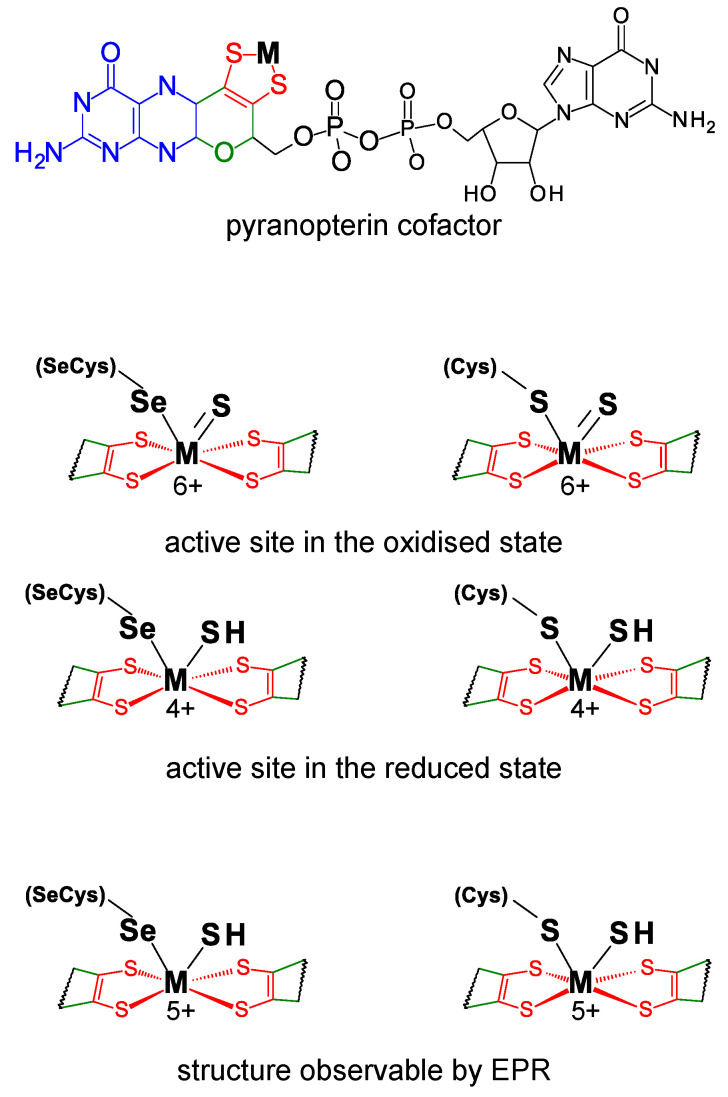
Active site structure of metal-dependent FDHs and FMFDHs. **Top**: Structure of the pyranopterin cofactor. The pyranopterin cofactor molecule is formed by pyrano(green)–pterin(blue)–dithiolene(red)–methylphosphate(black) moieties; in all so far characterized enzymes, the cofactor is found esterified with a guanosine monophosphate (dark gray). The dithiolene (–S–C=C–S–) group forms a five-membered ene-1,2-dithiolene chelate ring with the molybdenum or tungsten ion, here indicated as M (from metal). **Middle**: Structure of the active site in the oxidized and reduced state. **Bottom**: Active site structure supported by EPR data. In middle and bottom structures, for simplicity, only the dithiolene moiety of the pyranopterin cofactor is represented.

FDHs can be divided into two main classes. The metal-independent FDH class comprises enzymes, typically homodimers that have no metal ions or other redox-active centers, nor selenium [49,50,51,52,53,54]. These enzymes, found in bacteria, fungi, and plants, are NAD-dependent and belong to the *D*-specific dehydrogenases of the 2-oxyacids family. On the contrary, the metal-dependent FDH class, present only in prokaryotes, comprises enzymes that harbor different redox-active centers and display high structural diversity (Table 1) [41,42,43,45,46,48]. As the class name indicates, the active site of these enzymes holds one molybdenum or one tungsten ion in a very well conserved metal center (Figure 1). In its oxidized (6+) form, the metal (molybdenum or tungsten) is coordinated by the *cis*-dithiolene (–S–C=C–S–) group of two pyranopterin cofactor molecules, one terminal sulfido group (Mo^6+^/W^6+^=S), plus one selenium or one sulfur atom from a selenocysteine or cysteine residue (Mo^6+^/W^6+^-Se(Cys) or Mo^6+^/W^6+^-S(Cys)) (abbreviated as SeCys-Mo-FDH, SeCys-W-FDH, Cys-Mo-FDH, and Cys-W-FDH) [40,44,55,56]. Noteworthy, there is no apparent relation (as far as is presently known) between the metal (molybdenum or tungsten) and the presence of a selenocysteine or cysteine residue and catalytically efficient SeCys-Mo-FDH, SeCys-W-FDH, Cys-Mo-FDH, and Cys-W-FDH have been known for a long time.

Similar to FDHs, selenocysteine-containing and cysteine-containing *N*-formyl-methanofuran dehydrogenases (SeCys-FMFDH and Cys-FMFDH) exist and selenium’s role in FMFDH catalysis is unknown as well. FMFDHs are FDH-like enzymes that have two physically separated active sites: one catalyzes the reduction of carbon dioxide to formate, which is then intramolecularly transferred to the second active site, where it is condensed with methanofuran to form *N*-formyl-methanofuran [57,58,59,60]. The active site responsible for the carbon dioxide reduction is identical to the FDHs’ one and harbors one molybdenum or tungsten ion coordinated by the *cis*-dithiolene group of two pyranopterin cofactor molecules, one terminal sulfido group (Mo^6+^/W^6+^=S), plus one selenium or one sulfur atom from a selenocysteine or cysteine residue (Figure 1).

#### 3.1.2. Reaction Mechanism

**Figure 2:**
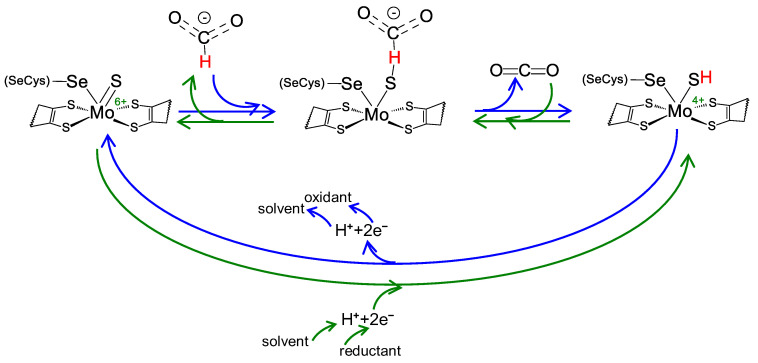
Reversible FDH and FMFDH reaction mechanism, as proposed by Maia et al. [62]. Reaction mechanism proposed for formate oxidation (blue arrows) and carbon dioxide reduction (green arrows) for both metal-dependent FDHs and FMFDHs. For simplicity, the mechanism is represented for a molybdenum, selenocysteine-containing enzyme, but it should be similar for tungsten and cysteine-containing enzymes. See text for details.

Regardless of the physiological function and structural complexity, the reaction mechanism of the interconversion of formate and carbon dioxide (Equation (1)) is believed to be similar in all these selenocysteine- and cysteine-containing FDH and FMFDH enzymes. As originally proposed by Niks et al. [61] for formate oxidation and shortly after also for carbon dioxide reduction by Maia et al. [62], it is currently well established that formate oxidation and carbon dioxide reduction proceed through hydride transfer, with the oxidized and reduced active site sulfido group, Mo/W^6+^=S and Mo/W^4+^-SH, acting as the direct hydride acceptor and donor, respectively (Figure 2) [63,64,65,66] (even though other atomic details of the reaction mechanism are not yet consensual; see, for example [67]). It is noteworthy that no direct role in the chemical transformations is presently ascribed to the selenocysteine or cysteine residue, in accordance with the existence of catalytically efficient SeCys enzymes and Cys enzymes (a similar situation occurs with molybdenum and tungsten). Nevertheless, it is expected that the presence of a selenocysteine or cysteine should affect the active site properties and that each enzyme type has to cope with the intrinsic differences between selenium and sulfur (see Section 3.3).

Briefly, formate oxidation (Figure 2, blue arrows) is initiated with the formate binding to the oxidized active site but not directly to the molybdenum/tungsten atom. Formate is suggested to bind in a binding pocket, where a conserved arginine residue “anchors” its oxygen atom(s) through hydrogen bond(s), and forces its Cα hydrogen to point towards the sulfido ligand (Mo^6+^/W^6+^=S). Subsequently, formate oxidation proceeds by a straightforward hydride transfer from formate to the sulfido group of the oxidized molybdenum/tungsten centre, leading to the formation of Mo/W^4+^-SH and CO_2_. The re-oxidation of Mo/W^4+^ to Mo/W^6+^ (via intramolecular electron transfer to the enzyme’s other redox center(s) and, eventually, to the physiological partner) and the release of carbon dioxide close the catalytic cycle. The now oxidized Mo/W^6+^ favors the sulfido group deprotonation (dictated by the ligand p*K*_a_ [68,69,70]) and the initial oxidized metal centre, Mo/W^6+^=S, is regenerated. Under non-steady-state catalytic conditions (such as the ones created in EPR experiments described below), the molybdenum/tungsten one-electron oxidation should be favored (Mo/W^4+^→Mo/W^5+^), leading to the formation of the EPR detectable species. 

The carbon dioxide reduction is suggested to follow the reverse reaction mechanism (Figure 2, green arrows) but starting with a reduced active site, holding a protonated sulfido group, Mo/W^6+^-SH (as is dictated by the ligands p*K*_a_ [68,69,70]). Carbon dioxide is suggested to bind to the same binding pocket, where the arginine residue is key to anchor it in the correct position to orient its carbon atom towards the protonated sulfido. Afterwards, the reaction proceeds through straightforward hydride transfer from the protonated sulfido group. This yields a formate moiety and Mo/W^6+^=S. The subsequent re-reduction of Mo/W^6+^ to Mo/W^4+^ (via intramolecular electron transfer from the enzyme’s physiological partner, through its redox center(s)) and formate release closes the catalytic cycle. The now reduced Mo/W^4+^ favors the sulfido group protonation and the initial reduced molybdenum/tungsten center, Mo/W^4+^-SH, is regenerated. 

### 3.2. How Was the Selenium Locus in Formate Dehydrogenases Established?

The presence and essentiality of selenium was demonstrated in pioneer works, mainly in the 1970s, following the incorporation in target enzymes of selenium-75 (present in the growth medium/feed). Actually, FDH was among the first enzymes shown to contain selenium [27,28]. 

The recognition of the presence of molybdenum or tungsten and selenium led to a series of spectroscopic studies that were decisive to the early characterization of the FDH active site. Electron paramagnetic resonance spectroscopy (EPR) was thoroughly explored (reviewed recently, for example, in [71,72,73]). In fact, the first evidence for the direct binding of selenium to a metal (molybdenum) active site center was obtained precisely with EPR [74,75]. The *E. coli* SeCys-Mo-FDH H was one of the first FDHs to be explored [75,76]. When reduced with formate, it gives rise to a nearly axial Mo^5+^ signal, with *g*_1_ = 2.094 and *g*_2,3_ = 2.001, 1.990, that displays coupling to one formate-derived solvent-exchangeable proton (*A*_1,2,3_(^1^H) = 7.5, 18.9, 20.9 MHz). When the EPR signal is generated from the ^77^Se-enriched enzyme, a very strong and anisotropic interaction is observed (*A*_1,2,3_(^77^Se) = 13.2, 75, 240 MHz [77], values that are almost five times higher than the ones observed in Mo-Se model compounds [77,78]). This strong interaction, observed simultaneously with the expected ^95,97^Mo hyperfine coupling, confirmed that the selenocysteine selenium atom is directly coordinated to the molybdenum (Figure 1) and suggested that the unpaired electron is delocalized 17–27% over the selenium (a finding in line with the expected covalency introduced by selenium in a Mo-Se bond) [77]. Moreover, photolysis assays showed that in the photo-converted enzyme, the interaction with ^77^Se is not significantly affected, while the interaction with the solvent-exchangeable proton disappears, thus providing further evidence that the selenium remains bound to the molybdenum during the catalytic turnover [77]. These photolysis assays were also key in providing additional evidence that the selenocysteine residue could not be the hydrogen atom acceptor during catalysis, as is currently accepted (Figure 2) [61,62]. (Note: Studies with ^2^H-labelled formate (in ^1^H-water) showed that the coupled solvent-exchangeable proton originates from the substrate molecule and that the proton acceptor is located within magnetic contact to the molybdenum center [77]. Similar results were obtained with *D. desulfuricans* [79], *D. vulgaris* [80,81,82,83], and *C. necator* [61] enzymes, overall suggesting that the hydrogen atom is transferred from formate Cα to the molybdenum center in the course of the reaction and then exchanged with the solvent. Hence, the current general consensus is that the structure of the EPR signal-giving species is a Mo^5+^-Se(Cys)(-SH) center that can arise from the one-electron oxidation/reduction of a catalytic intermediate (Figure 2) [61,62]).

These original studies with *E. coli* FDH H were supported and consolidated with other selenium-containing FDHs, including *Desulfovibrio desulfuricans* [79], *D. gigas* [84,85], *D. vulgaris* Hildenborough [80,81,82,83], and *Methylosinus trichosporium* [86] FDHs. These enzymes display rhombic Mo^5+^/W^5+^ EPR signals with small anisotropy, a well-resolved hyperfine structure due to ^95,97^Mo/^183^W, and interaction with a solvent-exchangeable proton (for example: *D. desulfuricans*: *g*_1,2,3_ = 2.012, 1.996, 1.985, *A*_1,2,3_(solvent-exchangeable ^1^H) = 23.1, 29.9, 27.8 MHz [79]; *D. vulgaris* Hildenborough FDH 1: *g*_1,2,3_ = 1.995, 1.881, 1.852, *A*_1,2,3_(^183^W)= 225, 129, 134 MHz [80]; *D. vulgaris* Hildenborough FDH 2 (main component): *g*_1,2,3_ = 1.982, 1.876, 1.902, *A*_1,2,3_(^183^W)= 232, 119, 151 MHz [82]). The *D. desulfuricans* FDH displays also a hyperfine interaction with a second non-solvent-exchangeable proton (*A*_1_ = 35.1 MHz, *A*_2,3_ not detectable) that was assigned to the metal-bound selenocysteine Cβ hydrogen atoms [79]. Together, the EPR data suggest an FDH active site holding a stable selenocysteine–metal ligation. It also suggests that the active site holds a transient proton-accepting site (within the metal magnetic contact) that was assigned as the terminal sulfido group (please see Note above) [61,62]. Overall, the EPR clearly points to the FDH active site having a Mo^5+^/W^5+^-Se(Cys)(-SH) structure (Figure 1), formed from one-electron oxidation/reduction of a catalytic intermediate (Figure 2) or by chemical reduction. 

The SeCys-FDH active site was also explored by X-ray absorption spectroscopy (XAS) since early times [87]. XAS at the molybdenum and selenium K-edges of the most explored model FDH, *E. coli* SeCys-Mo-FDH H, revealed four Mo-S ligands at 2.35 Å, one (originally not assigned) Mo=S at 2.1 Å, and one Mo-Se ligand at 2.62 Å, in both oxidized and reduced enzyme [88]. In the *D. desulfuricans* SeCys-Mo-FDH, the molybdenum and selenium K-edges data also showed a hexa-coordinated active site, with one Mo-Se ligand at 2.57 Å in both oxidized and reduced enzyme [89]. It is noteworthy that the replacement of the *E. coli* SeCys-Mo-FDH H selenocysteine by a cysteine residue abolished the Mo-Se fingerprint and gave rise to a spectrum consistent with five Mo-S ligands and one Mo=O at 1.7 Å [88]. Comparatively, XAS studies of native Cys-FDHs (for example, oxidized *Rhodobacter capsulatus* Cys-Mo-FDH [90,91]) confirmed that the cysteine residue is bound to the metal, as expected. Hence, the XAS results are in excellent agreement with the EPR proposed FDH active site structure, Mo^5+^/W^5+^-Se(Cys)(-SH) (Figure 1).

**Figure 3:**
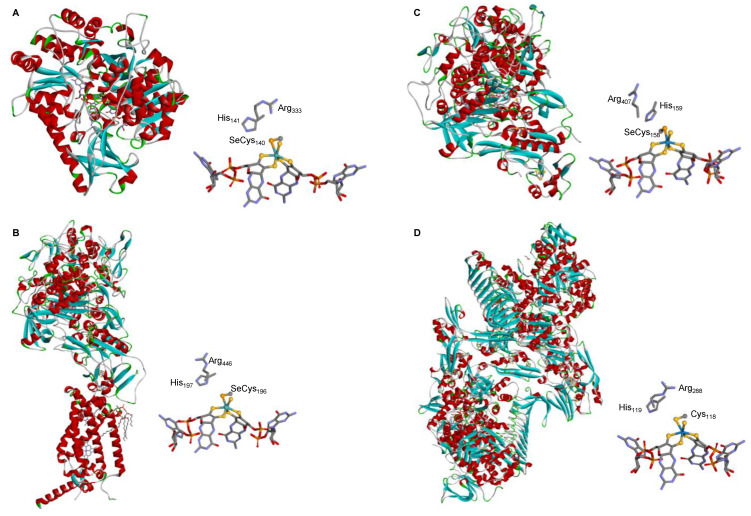
Three-dimensional structure view of some metal-dependent FDHs and FMFDHs and their active sites. (**A**) *E. coli* SeCys-Mo-FDH H [92]; (**B**) *E. coli* SeCys-Mo-FDH N [93]; (**C**) *D. gigas* SeCys-W-FDH [94]; (**D**) *M. wolfeii* Cys-W-FMFDH [59]. The structures shown are based on the PDB files 1FDO (**A**), 1KQF (**B**), 1H0H (**C**), and 5T5I (**D**) (α helices and β sheets are shown in red and cyan, respectively).

The crystallographic structure of different native SeCys- (and Cys-) FDHs entirely supports this active site structure. The first FDH 3D structure solved, in 1997, was the one of the model *E. coli* SeCys-Mo-FDH H [92] and this was the only one known for 5 years (2002), when the structure of two more enzymes were finally solved, the *E. coli* SeCys-Mo-FDH N [93] and *Desulfovibrio gigas* SeCys-W-FDH [94] (Figure 3). The first FMFDH structure (the *Methanothermobacter wolfeii* Cys-W-FMFDH) was revealed only 14 years after, in 2016 [59]. Presently, several structures are known [60,80,81,95,96,97,98,99] and the active site structure is firmly established to be the conserved Mo^6+^-Se(Cys)(=S), W^6+^-Se(Cys) (=S), Mo^6+^-S(Cys)(=S), or W^6+^-S(Cys)(=S) (Figure 1).

### 3.3. Why Do Some Formate Dehydrogenases Have a Selenocysteine and Not the Less “Expensive” Cysteine Residue?

Since its early identification as a selenium-containing enzyme, the role of selenium in FDH catalysis has intrigued the scientific community. A pioneer work in the late 1980s [100] with the model *E. coli* SeCys-Mo-FDH H showed that selenocysteine (SeCys_140_) replacement with a cysteine residue resulted in significant lower FDH activity, while replacement with a serine residue rendered the enzyme inactive. In a subsequent, more comprehensive work by the Stadtman group [101], it was clearly shown that selenocysteine replacement with a cysteine resulted in a marked decrease in FDH activity (*k*_cat_/*K*_m_^formate^ (SeCys-FDH) = 108 × 10^3^ M^−1^s^−1^ to *k*_cat_/*K*_m_^formate^ (Cys-FDH) = 1 × 10^3^ M^−1^s^−1^) and the Cys-FDH variant’s slower kinetics was suggested to be due to a lower rate of the hydrogen atom transfer step (deuterium (formate) isotope effect on *k*_cat_/*K*_m_). Simultaneously, the pH-dependent alkylation-induced inactivation of the native SeCys-FDH and variant Cys-FDH (reaction with iodoacetamide in the presence of formate) was shown to follow the trend of the expected p*K*_a_ values of each amino acid (native SeCys-FDH was inactivated more than 80% at pH > 6 (p*K*_a_ (SeCys) ≈ 5.2), while variant Cys-FDH was inactivated more than 80% only at pH > 7 (p*K*_a_ (Cys) ≈ 8.2). Together, these results were taken to suggest that selenol (versus thiol) plays an essential role in catalysis. However, both native SeCys-FDH and variant Cys-FDH followed the same kinetic mechanism (ping-pong, bi-bi) and displayed similar pH dependencies with respect to activity and stability, which makes it difficult to reconcile with the hypothesis that a cysteine residue would render a catalytically incompetent enzyme because of its thiol features. 

As other variant enzymes are studied, it is becoming clear that it is not surprising that variants are less active than wild types. Most relevant to the present discussion was the recognition that several “wild-type variants” (native Cys-FDH) exist that are as catalytically efficient as the native SeCys-FDHs (Table 1). In fact, several native Cys-FDHs were known for long, but they were overlooked by the groups studying FDH catalysis, which focused instead on a few model enzymes, mostly in *E. coli* SeCys-Mo-FDH H. In addition, coincidentally, those FDHs whose 3D structures were first solved (see Section 3.2) were all SeCys-FDHs and, thus, selenium acquired a highlighted role in FDH catalysis that is not consistent with the existence of native Cys-FDHs.

Presently, the accepted FDH reaction mechanism does not ascribe any direct role to the selenocysteine or cysteine residue (see Section 3.1.2), leaving open the question of why some formate dehydrogenases have a selenocysteine and not the common cysteine residue.

Selenocysteine incorporation is highly demanding (“expensive”) for the cell. It requires additional energy and dedicated machinery to uptake selenium and to synthesize and orchestrate different biomolecules that lead to the recognition of target UGA-codon by specific *t*RNA molecules (and not as the “opal” stop-codon), culminating in selenocysteine being incorporated in the target protein [102,103,104,105,106]. Therefore, it is generally accepted that the presence of selenocysteine should constitute an intrinsic advantage for the cell [14,107,108,109]. Regarding FDHs, such an advantage was not yet proven. Among other hypothesis, we can think (as for other proteins, for example [110,111]) that the comparatively higher difficulty in forming higher oxidation states of selenium and the higher facility to non-enzymatically reduce them is an advantage for those organisms whose lifestyle makes their FDHs more prone to suffer oxidative modifications. 

Regardless of the biological pressure behind the evolution of native SeCys-FDHs and native Cys-FDHs, it should be kept in mind that selenium is not a sulfur (see Section 2). Thus, it is reasonable that the presence of one or the other alters the reaction energy pattern, in spite of both enzyme types operating through the same general hydride transfer mechanism (same chemical transformations). Therefore, in order to be catalytically efficient, each enzyme type should have evolved a strategy to compensate for those Se/S physicochemical differences. Hence, more interesting and relevant than studying why some FDHs have selenium is to understand the strategies that allow both SeCys-FDH and Cys-FDH to be catalytically efficient. For example, it must be understood how the Cys-FDHs compensate for the presence of a less covalent Mo-S(Cys) bond, or, depending on the origin, how the SeCys-FDHs compensate for the more covalent selenium, because those Se/S-metal bond features are expected to influence the metal center reduction potential, which, in turn, modulates the electron transfer process (a step that even though it is not a chemical transformation is decisive for catalysis). 

## 4. Hydrogenases

Hases are crucial role as an alternative energy source as they have potential applications in green hydrogen production [112,113]. Hydrogenases are a heterogeneous group of enzymes that differ in size, subunit composition, metal content, and cellular location (periplasmic, cytoplasmic, and cytoplasmic membrane-bound) and catalyze the reversible two electron oxidation of hydrogen (Equation (2)).

H_2_ ⇌ 2H^+^ + 2e^−^ (2)

### 4.1. Enzymatic Machineries

**Figure 4:**
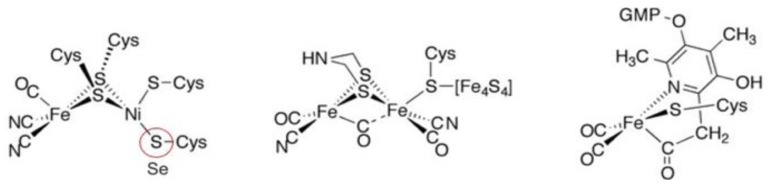
Active site structure of [NiFe]-, [FeFe]-, and [Fe]-hydrogenases [112].

**Figure 5:**
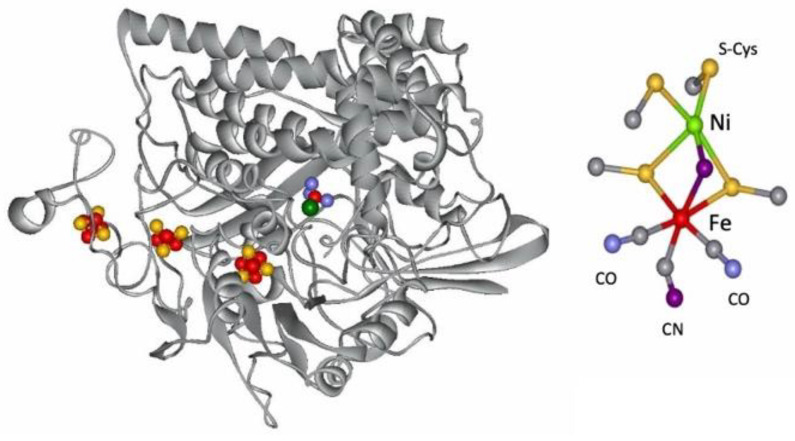
Structure of the *D. gigas* hydrogenase enzyme and of its active site.

The metal-containing hydrogenases are subdivided into three classes: [Fe]-, [FeFe]-, and [NiFe]-hydrogenases (Figure 4) [112,113,114,115,116,117]. [Fe]-hydrogenases only contain one Fe ion in their active site and are designated as “Fe-only” hydrogenases. [FeFe]-hydrogenases contain an unusual iron-sulfur cluster termed the H-cluster that consists of an [Fe_4_S_4_] subcluster bridged via a cysteine (Cys) thiolate to the binuclear iron subcluster, also coordinated by inorganic ligands: two S atoms and one CO or CN ligand. [NiFe]-hydrogenases are heterodimeric proteins constituted by a small and a large subunit (Figure 5). The small subunit accommodates three iron-sulfur clusters (two [4Fe-4S] clusters and one [3Fe-4S] cluster) involved in the electron transport to/from the active site ([NiFe] cluster); the large subunit contains the catalytic site: the nickel-iron center. In some [NiFe]-hydrogenases, one of the Ni-bound cysteines is replaced by a selenocysteine, and [NiFe]- and [NiFeSe]-hydrogenases represent a single superfamily, and the Ni-Fe core contains unusual ligands: carbon monoxide (CO) and cyanide (CN^−^).

The [NiFe-Se] hydrogenases are found in some species of *Desulfovibrio* sp. The genes encoding the large and small subunits of the periplasmic hydrogenase from *Desulfovibrio (D.) baculatus* (DSM 1743) exhibit homology (40%) to the [NiFe] hydrogenases. The gene for the large subunit contains a codon (TGA) for selenocysteine in a position homologous to a codon (TGC) for cysteine in the [NiFe] hydrogenase. Spectroscopic studies support that selenium is a ligand to the nickel site (see below) [118,119,120,121,122,123].

**Figure 6:**
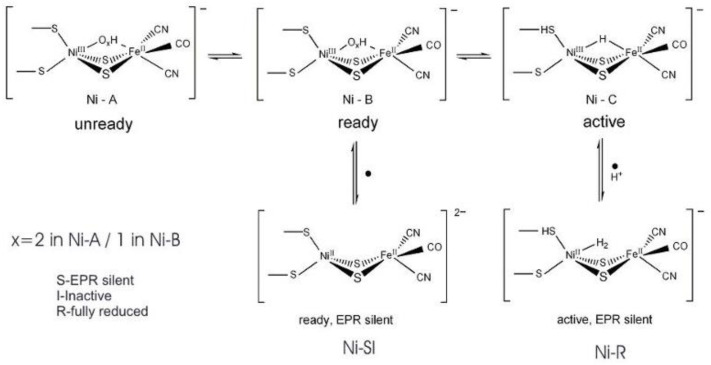
Redox and catalytic intermediates in [NiFe] hydrogenases. Adapted from [131].

As isolated, the active [NiFe] cluster contains a Ni(III) and a low-spin Fe(II) (diamagnetic) that remain unchanged during the enzyme mechanism. Different oxidized inactive states are attained by the enzyme. In general, the isolated states are mixtures of “unready” Ni-A and “ready” Ni-B states (Figure 6). These states show delocalized electron density between nickel and iron, attributed to a third bridging oxygenated ligand. Both oxidized states are paramagnetic and characterized by different EPR g-values. The bridging ligand in the Ni-B state has been assigned to an OH^−^ ligand and a water molecule is probably present in the Ni-A state [124,125,126,127]. After the reaction with the substrate (hydrogen), (Ni-C) develops with a bridging hydride (H^−^) ligand. Other intermediates were denominated Ni-R and Ni-SI. In all states of standard hydrogenases, the nickel atom has a vacant or labile coordination site and, therefore, Ni represents the primary hydrogen binding site. The H_2_ molecule can be accessed by the buried [NiFe] active site through hydrophobic tunnels leading to the Ni atom [128,129,130,131].

The Ni site, in the [NiFeSe] cluster, is coordinated by three sulfur atoms from three cysteine residues and one Se atom from selenocysteine. The electron transfer pathway is similar to one described in [NiFe] enzymes, involving three iron–sulfur clusters present in the small subunit connecting the active site to the surface; however, the medial cluster is a [4Fe-4S] cluster instead of the [3Fe-4S] cluster present in [NiFe] hydrogenases [132,133,134,135,136]. 

The role of the selenocysteine has a remarkable influence on the catalytic properties of [NiFeSe] hydrogenases: (i) high catalytic activity in H_2_ production direction is detected and is less sensitive to oxygen [118,137,138,139]; (ii) in general, the as-purified [NiFeSe] hydrogenases are almost EPR silent (Ni-A and Ni-B signals are not or are weakly detected). Upon reduction, the Ni-C EPR signals, assigned to active states of the fully developed enzyme, with spectral characteristics as observed in [NiFe] hydrogenases [135,140,141]; (iii) different oxygen permeation pathways in [NiFe] and [NiFeSe] hydrogenases have been described, based on computational studies [142]. 

### 4.2. Selenium and the Hydrogenase Reaction Mechanism

**Figure 7:**
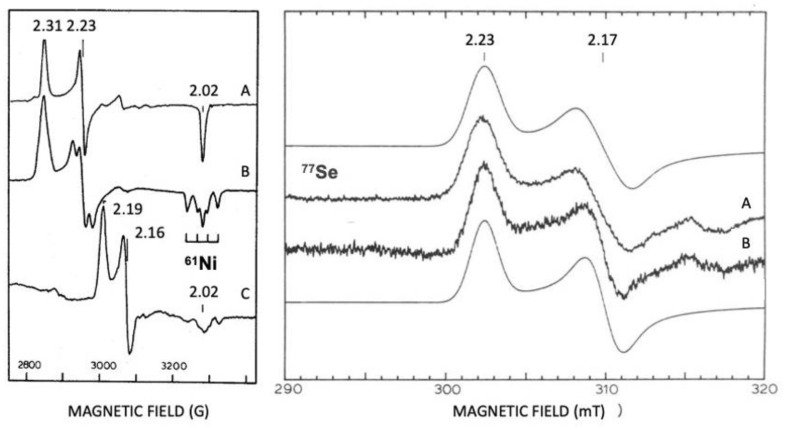
Revealing EPR, Ni, and Se at the active site of hydrogenases. Isotopic substitutions with ^61^Ni and ^77^Se. Left panel *D. gigas* [Ni-Fe] Hase. (**A**) Ni-A ^61^Ni un-enriched; (**B**) Ni-A ^61^Ni enriched; (**C**) Ni-C ^61^Ni enriched. Right panel *D. baculatus* [Ni-Fe-Se] Hase. (**A**) Ni-C ^77^Se enriched and (**B**) Ni-C ^77^Se un-enriched; mooth lines are simulations of spectra A and B. Adapted from refs [143,145].

Isotopic substitutions are crucial for the identification of Ni and Se in hydrogenases. A ^61^Ni isotope was used for assigning EPR signals to Ni (Figure 7) [143]. Selenium contains six isotopes, and five of them are stable (atomic numbers 74, 76, 77, 78, and 80). The sixth isotope, with an atom abundance of 8.73%, is selenium-82 (^82^Se), a beta emitter which is weakly radioactive. The ^77^Se isotope (7.5%) is a useful EPR marker, with an I = 1/2. ^33^Se and ^77^Se are useful markers for spectroscopic studies (EXAFS and EPR) (Table 2) [143,144,145,146]. 

**Figure 8:**
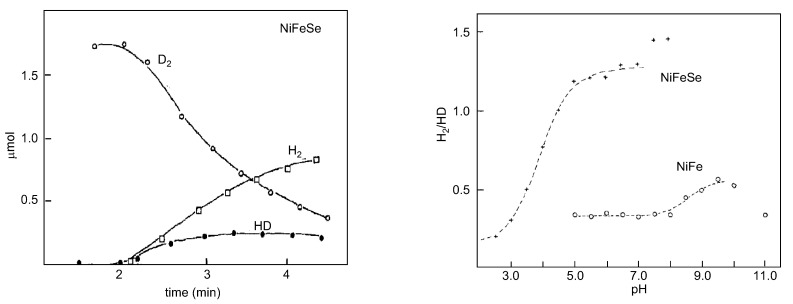
D_2_/HD exchange activity of *D. salexigens* [NiFeSe] hydrogenase (**left panel**) and variation in the experimental ratios H_2_/HD as a function of pH (**right panel**) of *D. baculatus* (cytoplasmic) [NiFeSe] hydrogenase and *D. gigas* [NiFe] (periplasmic). Left panel adapted from [140]; right panel adapted from [141].

Proton–deuterium exchange measurements are quite appropriate to probe the influence of the Se–cysteine ligand in the mechanism of hydrogen handling. An important clue was the observation that the H_2_/HD ratios were higher for [NiFeSe] hydrogenases than those observed for the [NiFe] ones, which is related to the activation of the hydrogen molecule (Figure 8).

Several studies on the role of transition metals in hydrogenation reactions describe the main processes for the activation of the H_2_ molecule, catalyzed by transition metals, and the hydride–metal complex (rarely detected) has been indicated to be involved, with evidence mostly supporting kinetic studies of the reactional mechanisms involved [118,135,141,147].

oxidative addition: M^n+^ + H_2_ ⇌ M^n+^ H_2_ (3)

homolytic cleavage: 2 M^n+^ + H_2_ ⇌ 2 M^n+1^ H (4)

heteroliyic cleavage: M^n+^ + H_2_ ⇌ M^n+^ H^−^ + H^+^ (5)

The exchange reaction with D_2_/H^+^ or H_2_/D^+^ gave important clues and was studied using whole cells, crude extracts, and purified enzymes, supporting the heterolytic cleavage mechanism since the first product of the reaction is HD. Also, by thermodynamic arguments, the heterolytic cleavage is favored in the homolytic process [148]. Isotopic exchange between D_2_ and H^+^ and the *ortho*/*para* hydrogen conversion is also consistent with the heterolytic cleavage of the hydrogen molecule. The presence of a metal–hydride complex and of a proton acceptor site for the stabilization of the proton by a base (external or a metal ligand) is a necessary requirement, as indicated in Equations (6) and (7) [118,135,141,147].

M + H_2_ + B ⇌ M–H^−^ + B–H^+^ (6)

M...X + H_2_ ⇌ M–H^−^ + X–H^+^ (7)

On the basis that the enzyme-bound H or D atoms exchange more rapidly with the solvent than the hydride, HD is the initial product, but D_2_ (or H_2_) is, however, the final product of the total exchange process since there occurs a secondary exchange step of the HD molecule. If the hydride and proton acceptor sites can exchange independently with the solvent, the amount of HD and D_2_ produced depends on the relative exchange rates of both sites and, consequently, the ratio of products should be pH-dependent (as supported by the available experimental data). In reality, the alteration in the p*K*_a_ values of the proton acceptor at the active site will be reflected in the isotope ratios [149,150,151]. The [NiFeSe] hydrogenases have H_2_/HD ratios greater than 1 (Figure 8). The [NiFe] hydrogenases isolated from *D. gigas*, *D. multispirans* n.sp., and *D. desulfuricans* (ATCC 27774) show a ratio of H_2_/HD smaller than 1 (0.3) at pH 7.6, but maximal activity is generally attained at intermediate pH values. This trend is further evidence that a heterolytic process is operative by analogy with inorganic models such as the (Pd-salen) complex [151]. *D. baculatus* and *D. gigas* hydrogenases show pH-dependent H_2_/HD ratios. The rate-limiting step for the cleavage process at acidic pH values is the protonation of the proton-accepting site. At basic pH values, the liming step is the reformation of the H_2_ molecule since the proton-accepting site has been deprotonated [141,142]. The curve follows the profile of a normal titration curve reflecting the protonation of the proton acceptor site. In the pH range 5–11, the H2/HD ratio is always smaller than 1 for the *D. gigas* enzyme. The same ratio calculated for a *D. baculatus* cytoplasmic hydrogenase is greater than 1 at pH *>* 5. Substitution of one of the sulfur ligands to the nickel by the less electronegative selenium may have a direct effect on the destabilization of the hydride form of this hydrogenase.

### 4.3. Overview

Hydrogenases are a clear case study of the influence of Selenium (as a Se-Cys) on the modulating or fine-tuning of enzyme catalytic properties through an acid-base equilibrium at the proton acceptor site or at the hydride site and should be explored for protein design and molecular modelling [117]. 

The [NiFeSe] hydrogenases clearly emerge as a subgroup of [NiFe] and there is a structural homology between [NiFe] and [NiFeSe]. However, [NiFeSe] is distinct in terms of its catalytic and active-site composition. Electrochemical studies help to reveal the interplay between the catalytic intermediates [152]. These enzymes display very interesting catalytic properties for biological hydrogen production and bio-electrochemical applications: high H_2_ production activity, low H_2_ inhibition, and O_2_ tolerance [153].

The direct role of selenocysteine in [NiFeSe] hydrogenase maturation and catalysis has also been discussed. An expression system for the production of recombinant [NiFeSe] hydrogenase from *Desulfovibrio vulgaris* Hildenborough and study of a selenocysteine–to-cysteine variant (Sec489Cys) in which, for the first time, a [NiFeSe] hydrogenase was converted to a [NiFe] type, reveal the direct involvement of this residue in the maturation process. It was proposed that selenium plays a crucial role in protecting against oxidative damage and the high catalytic activities of [NiFeSe] hydrogenases [133].

## 5. Glutathione Peroxidases

**Figure 9:**
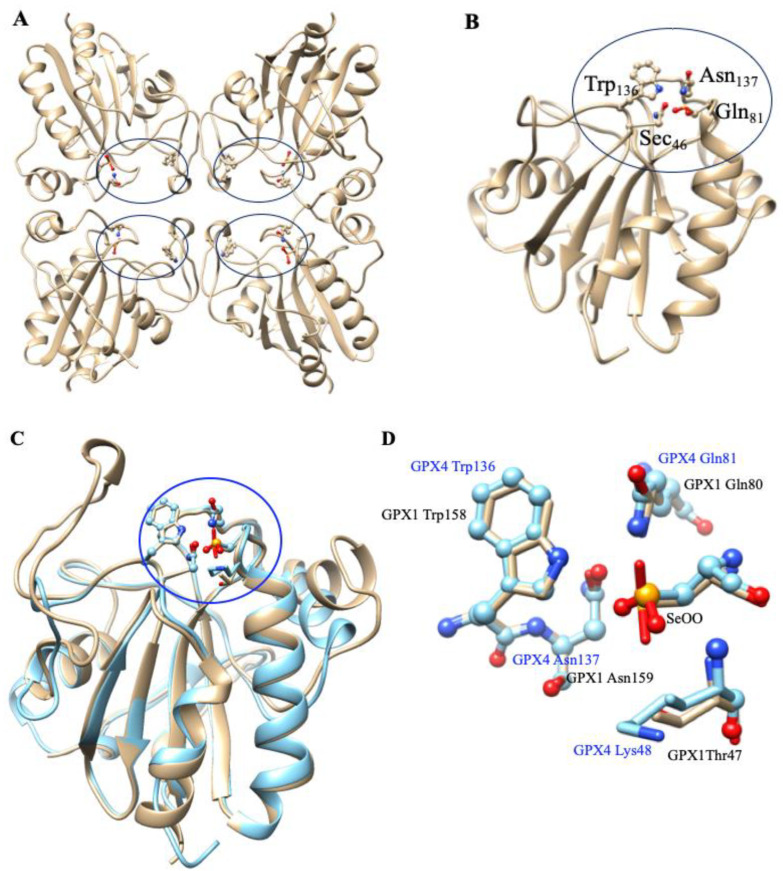
Crystal structure of GPxs. (**A**) Homo-tetramer of GPx1 (PDB file 1GP1) and (**B**) monomer of GPx4 (light blue; PDB file 6ELW). (**C**) Superimposed image of the crystal structures of GPx1 (one sub unit) and GPx4. (**D**) Highlighted is the conserved tetrad in the catalytic cycle of GPx1 and GPx4.

GPx is a multiple-isozyme family which protects the cellular organism from oxidative stress by the reductive transformation of hydroperoxide (H_2_O_2_) or organic hydroperoxide substrates (ROOH) to the product of H_2_O or alcohol, respectively, using cellular glutathione (GSH) as an electron source [154,155]. In 1952, Mills and Co-workers first noticed that GP_X_ protected hemoglobin from oxidative degradation [156]. After that, in the 1960s, GP_X_ activity was also observed in the lungs and kidneys [157]. In the 1970s, GPx was characterized and discovered selenocysteine amino acid, which played a vital role in enzymatic activity [158,159,160]. In the GPx family, only one GPx_1_ member was known until the 1980s. Then, this family grew to eight members [161]. In humans, five GPxs (GPx_1–4_ and GPx_6_) are encoded with selenocysteine residue in their catalytic site, whereas the rest (GPx_5_, GPx_7_, and GPx_8_) contain conventional Cys residue in their catalytic site [154,162,163,164,165]. The active site of GPxs possesses a conserved tetrad that is constructed by four amino acid residues including glutamine (Gln), asparagine (Asn), tryptophan (Trp), and either cysteine (Cys) or selenocysteine (Sec) [166,167]. For instance, the catalytic tetrad site of human GPx_4_ possesses Sec_46_, Gln_81_, Trp_136_, and Asn_137_ residues. The catalytic site is normally present at the *N*-terminal (Figure 9) [168]. The crystal structures of GPx_1–3_ and GPx_6_ are homotetrameric enzymes with masses of ~22–25 kDa in each subunit, whereas GPx_4_ is a monomeric enzyme with a mass of ~20–22 kDa (Figure 9) [169,170]. 

**Figure 10:**
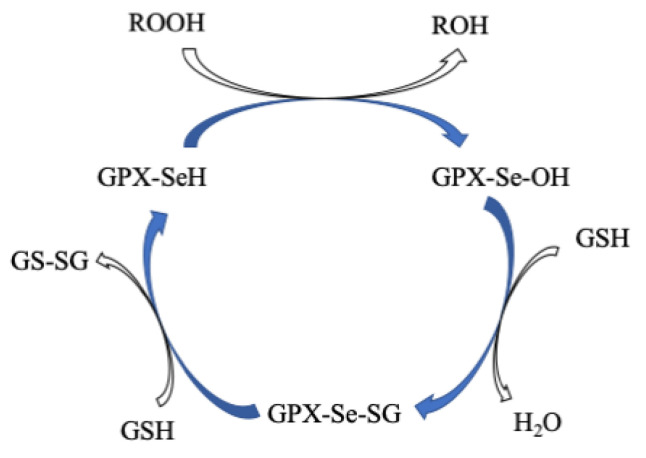
Proposed catalytic cycle of GPxs. Modified from [156].

All GPxs display two steps of redox reactions in their catalytic cycle (Figure 10) [171,172]. In the first step, the selenocysteine (Sec-SeH) is oxidized to selenic acid (Sec-SeOH), which is a key intermediate product in the catalytic cycle. Simultaneously, the toxic hydroperoxide is reduced to the corresponding alcohol. In the second step, the reduction of oxidized Sec-SeOH proceeds into two subsequent 1 e^−^ reduction steps. The Sec-SeOH is converted into GPx-SeGS by interacting with one equivalent reduced GSH, followed by the reduction of GPx-SeGS into GPx-Se by a second equivalent GSH for the next catalytic cycle [156,158,173,174,175]. The intermediate Sec-SeOH is stabilized by Gln and Trp, which are in the catalytic tetrad site [170], and additional Asn in tetrad contributes to the catalytic reaction [167]. Interestingly, the further oxidation product of Sec-SeH is seleninic acid (SeOO^−^), which is found in the crystal structure of GPx4 (Figure 9), suggesting that selenium can shuttle between selenenic acid (RSeO^−^) and seleninic acid (R-SeOO^−^) redox states in the extended catalytic cycle. The highly oxidized R-SeOO^−^ state in the enzyme may revert to the initial reduced state, R-Se^−^ via RSeO^−^, if suitable reducing species are available. This result may conclude that in a cellular redox state, the catalytic cycle of GPx4 may be mainly involved in R-Se^−^ and R-SeO^−^ redox states (low-oxidation cycle), but under oxidative stress, the catalytic cycle of GPx4 may be involved in R-SeO^−^ and R-SeOO^−^ redox states (high-oxidation cycle) [168]. The high-oxidation catalytic cycle may revert to a low-oxidation catalytic cycle if oxidative stress is overcome to the cellular redox state. 

## 6. Thioredoxin Reductases

TrxR belongs to the pyridine nucleotide–disulfide oxidoreductases family, of which some members are glutathione reductase, mercuric ion reductase, and lipoamide dehydrogenase [176]. A homodimeric flavoenzyme, it contains one redox-active dithiol/disulfide motif, FAD prosthetic group, and an NADPH binding site in each monomeric subunit [177]. TrxRs are distributed in all living systems and are generally classified into two major classes: (a) low molecular weight (LMW~35 kDa) TrxRs that are present in both lower eukaryotes and prokaryotes, and (b) high molecular weight (HMW~55 kDa) TrxRs that are present in higher eukaryotes [176,178]. Both classes of TrxR utilize NADPH as an electron source to reduce the oxidized state of TrxR that plays a vital role in cell proliferation. Due to large differences in structures, both classes of TrxRs have different catalytic paths to execute the same biochemical reaction. The LMW TrxRs have two redox centers such as an *N*-terminal dithiol/disulfide pair and an FAD prosthetic group [179,180], whereas HMW TrxRs contain three redox centers such as an *N*-terminal dithiol/disulfide pair and an FAD prosthetic group and sixteen additional amino acid residues with penultimate selenocysteine (Sec) in the catalytic site (-Cys-Secys-Gly sequence) at the end of the *C*-terminal [181,182,183,184].

**Figure 11:**
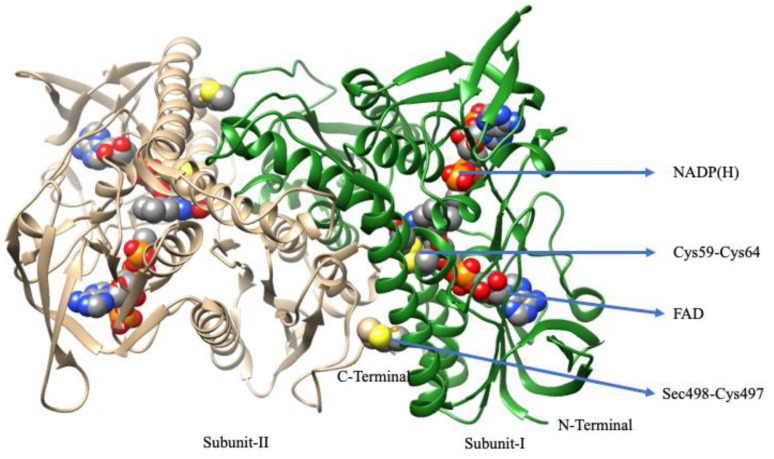
Crystal structure of dimer (subunit-I (green) and subunit-II (grey)) of recombinant rat selenocysteine TrXR 1 (PDB file 3EAO); highlighted are the redox centers in each subunit: *N*-terminal redox centre (Cys64 andCys59), FAD domain, and *C*-terminal redox centre (SeCys497 and Cys498).

There are three types of Mammals’ TrxRs: (a) the cytosolic form, TrxR1 [185], (b) the mitochondrial form, TrxR2 [186,187], and (c) the testis-specific thioredoxin glutathione reductase (TGR) [188]. The overall protein fold of TrxR1 [189] is similar to other TrxR2 [190] and TGR [191]. Among them, TrxR1 is well-characterized. In 2001, the first three-dimensional (3D) structure of rat TrxR1 (Sec to Cys mutant) [189], followed by a large number of 3D structures (Sec-substituted mutants) of human TrxR1 [192] and mouse TrxR2 were published [190,193]. In 2009, the crystal structure of recombinant rat TrxR1 with Sec amino acid was reported by Cheng et al. [194]. However, the overall structure of rat TrxR1 is similar to human TrxR1. The 3D structure of rat TrxR1 reveals that it is a homodimeric protein and the two subunits arranged in a head-to-tail manner and each subunit consist of three domains which are the *N*-terminal, *C*-terminal, and interface domain. The *N*-terminal harbors FAD, NADPH, and the dithiol redox center (Cys59 and Cys64), and the *C*-terminal harbors a flexible sixteen amino acid extension with the selenolthiol redox centre (Cys497 and Secys498) (Figure 11) [194]. The two redox centers are far from to each other, but for the activity of TrXR, they come close to each other, forming a dimeric species where the *C*-terminal redox centre of one subunit directly interacts with the buried *N*-terminal redox centre of another subunit. 

**Figure 12:**
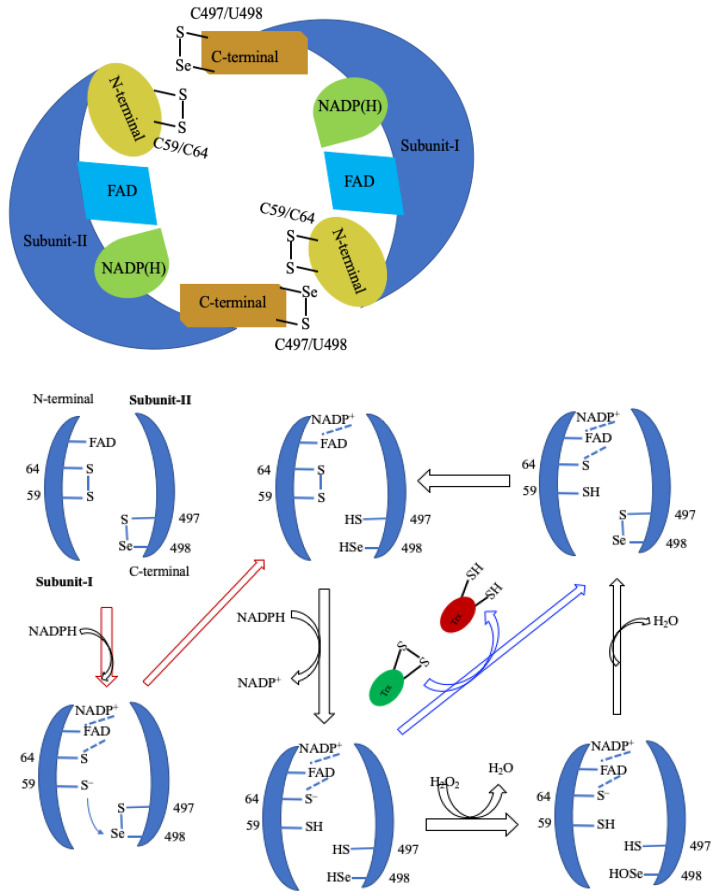
(**top**) Cartoon represents head-to-tail model of rat TrxR1 and (**bottom**) simplified possible mechanism for H_2_O_2_ or Trx reduction by TrxR. Modified from [196,197].

TrxR is an important biological redox mediator for the two-electron reduction of substrates. The catalytic cycle of mammalian TrxR involves three redox centers: *N*-terminal dithiol (Cys_59_–Cys_64_), adjacent FAD/NADH, and *C*-terminal selenolthiol pair (Cys_498_–Sec_497_) in the other subunit), which relay e^−^ from *N*-terminal dithiol to the substrate, thioredoxin via FAD/NADH. The human TrxR1 substrate–thioredoxin (Trx) complex is identified and the 3D structure of that complex reveals that the *C*-terminal arm binds with the substrate Trx through the disulphide bond (TrxR-Cys-S-S-Cys-Trx) [195]. A proposed mechanism of TrxR with Trx or small substrates (H_2_O_2_) is shown in Figure 12. The catalytic cycle starts by the 2e reduction of the Sec-Se-S-Cys to selenolate anion (Sec-Se^−^) that reduces the Trx or substrate (like H_2_O_2_). For reduction, Cys-Se-S-Cys gains 2e electrons from NADPH via the FAD–dithiol (Cys_59_–Cys_64_) complex to produce Cys-SH and Sec-Se^−^ at the *C*-terminal redox centre. The Sec-Se^−^ ion is a relatively strong nucleophile over Cys-S^−^. Therefore, Sec-Se^−^ is more susceptible to oxidized selenenic acid (-SeOH) by H_2_O_2_ compared to Cys-S^−^. Once it is formed, the adjacent cysteine thiol (Cys_497_) reacts with selenenic acid to yield H_2_O and selenenylsulfide that regenerates for the next cycle [196,197]. A similar catalytic mechanism is observed with Trx. The Sec-Se^−^ nucleophilic attacks on the disulfide bond of oxidized Trx, yielding an enzyme–Trx complex through the selenenylsulfide bond, which is reopened by attacking the Cys497 of the selenolthiol pair (Cys_498_–Sec_497_), and subsequently forming selenenylsulfide (Sec498-Cys497) [196,198].

## 7. Iodothyronine Deiodinases

**Figure 13:**
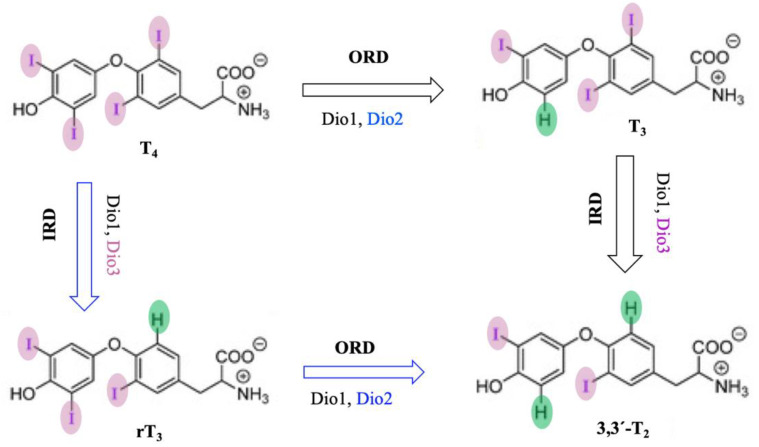
Probable mechanism of deiodination by deiodinase with thyroid hormone substrates.

Dios are selenocysteine-dependent mammalian deiodinase enzymes that regulate thyroid hormones by deiodination of iodothyronine [199,200,201,202]. Dios have been classified into three isoforms, Dio1, Dio2, and Dio3, based on their sequence of amino acids and specificity of substrates. Dio1 enzymes non-selectively catalyze both inner- (phenolic group) and outer-ring (tyrosine group) deiodination of thyroid hormones, but Dio2 and Dio3 both selectively catalyze outer-ring and inner-ring deiodination (ORD and IRD) of thyroid hormones, respectively (Figure 13) [203,204,205,206,207,208,209]. For instance, Dio1 catalyzes the conversion of pro-hormone thyroxine (l-3,5,3′,5′-tetraiodothyronine; T4) to the biologically active hormone 3,5,3′-triiodothyronine (T3) or 3,3′,5′-triiodothyronine (rT3) by eliminating one iodine atom from ORD or IRD [210,211,212,213], whereas Dio3 (or Dio2) converts T3 (or rT3) into the biologically inactive hormone, 3,3′-T2. Therefore, Dio3 plays a vital role in protecting the cells from elevated thyroid hormones [203,204,205,206,207,208,209,214,215]. 

## 8. Selenoproteins and Human Health

Selenoproteins (SePs) have been associated with many human health benefits but dysfunction of these proteins is associated with various human diseases such as diabetes, cancer, and viral infections [8,216,217,218]. SePs, particularly GPxs and TrxRs enzymes, participate in redox homeostasis and are believed to be a main contributing factor in the development and progression of various disease states [216]. 

### 8.1. Cancer

Compared to healthy cells, cancer cells generally harbor elevated reactive oxygen species (ROS), causing their abnormal growth with a high metabolic rate. To adjust the redox balance, cancer cells upregulate antioxidant systems to cope with the elevated ROS [197,219]. GPxs and TrxRs both can protect cancer cell development and progression by their antioxidant roles. 

To date, several studies have attempted to analyze the role of GPxs, as well as changes in GPxs levels, in different types of tumors [216,220], but it remains controversial [221]. Indeed, GPx1 inhibits the oxidation of DNA mutations and, therefore, it may inhibit tumorigenesis [222], and overexpressed GPx1 reduces tumor growth, suggesting its protective effect in tumorigenesis [223]. However, reduced expression of GPx1 is detected in thyroid cancer [224], gastric cancer [225], and colorectal cancer [226], whereas GPx1 is highly expressed in kidney cancer [227] and pancreatic cancer [228]. Similar to GPx1, unusual expression of GPx2 is also observed in different tumors; for example, GPx2 is overexpressed in colorectal cancer [229], whereas a lower expression of GPx2 is detected in prostate intraepithelial neoplasia [230,231]. Regarding GPx3, it can be considered a novel tumor-suppressor gene [232,233] because hypermethylation is detected with down-regulation of GPx3 in tumor patients with Barrett’s esophagus [234], prostate cancer [235], and endometrial adenocarcinoma [232,236]. Like GPx1-3, GPx4 is also a tumor suppressor due to its down-regulation in breast cancer [237] and pancreatic cancer [223]. In addition, overexpression of GPx4 reduces fibrosarcoma cell growth [238]. The role of other GPxs in tumorigenesis still remains controversial due to limited research [221].

Importantly, in excess, GPx may have detrimental effects due to a lack of necessary cellular oxidants [239,240] that can respond to cell growth, mitochondrial function, disulfide bond formation in protein, and cellular metabolism [241,242,243,244]. As GPx-1-4 are selenoproteins, these are readily affected by selenium levels in the cell. Several studies have shown that mixed results are observed in cancer after the administration of selenium supplements; therefore, selenium supplementation has a complex effect [245,246,247]. 

Polymorphism of human GPxs gene is a common phenomenon and it is associated with various diseases, especially tumors [248]. The GPx1 gene has various genetic polymorphisms and its most common polymorphism is the substitution of cytosine (C) to thymine (T) in DNA, resulting in the alteration of amino acid from proline (Pro) to leucine (Leu); thereby, the activity of GPx1 reduces by 5% [249]. Pro198Leu GPx1 polymorphism is associated with various types of cancer, mainly breast [250], prostate [251], lung [252], bladder [253], leukemia [254], and colon cancers [255]. However, the connection between GPx1 polymorphism and cancer vulnerability is controversial and inconclusive.

**Figure 14:**
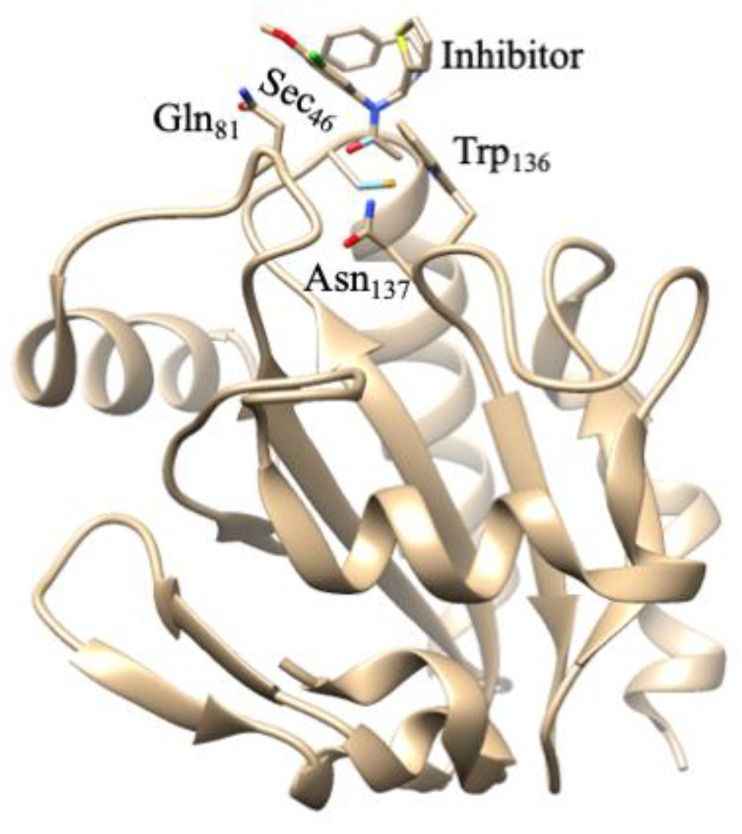
Crystal structure of human GPx4 with ML162 (inhibitor). PDB file 6HKQ.

However, GPxs are overexpressed in several types of cancer/tumor cells and act as tumor promoters. Therefore, many studies are devoted to reducing the activity of GPxs by using suitable inhibitors for cancer therapy. Interestingly, several studies describe that the inactivation of GPx4 by the inhibitor of ferroptosis leads to oxidative destruction of the cancer cell via ferroptosis [256,257,258]. Therefore, GPx4 is considered to be a potential cancer therapy target. Several small-molecule drugs have been recognized as inhibitors of GPx4 that were originally pointed out as a modulator of ferroptosis in cancer/tumor cells. These small-molecule drugs are RSL3 [259], ML162, and ML210 [260]. The crystal structure of human GPx4 with an ML162 inhibitor (S enantiomer) (Figure 14) [261] reveals that ML162 is covalently bonded at the active site of GPx4, thus resulting in inactivation of the enzyme. GPx4 contains a selenocysteine in the catalytic site that affects redox regulation by consuming ROS [168]. Overall, GPxs have a dichotomous role as a tumor/cancer suppressor and in cancer progression. Therefore, more studies are needed to understand the dichotomous roles of GPxs in cancer.

Similar to GPxs, elevated TrxR levels are associated with the progression of tumor cells and increasing tumor drug resistance [197,262]. Several studies have reported that high levels of TrxR are observed in several human cancer cells, like the human A549 lung cancer cell line; thus, inhibiting TrxR function may be a promising strategy for cancer/tumor therapy [263,264,265,266,267]. TrxR contains two catalytic sites: –Cys_497_–Sece_498_– and –Cys_59_–Cys_64_– which reduce ROS, thus inhibiting its catalytic activity to halt cancer proliferation [268,269,270,271]. Therefore, several TrxR inhibitors have been reported to be anticancer agents and these are under pre-clinical and clinical trials [272]. Indeed, auranofin, a gold phosphine compound, can inhibit the catalytic activity of TrxR via interaction with a Sec amino acid residue at the catalytic site [271,272,273,274,275].

### 8.2. Diabetes

Diabetes mellitus (DM), a common human health problem around the globe, is a metabolic disorder and it is characterized by high levels of blood sugar (hyperglycemia), causing dysfunction in insulin secretion and/or sensitivity [276,277,278,279,280]. Insulin is a hormone synthesized in the β-cell of the pancreas and its action is also regulated by the pancreatic β-cell [280]. The most common type diabetes is Type 2 diabetes mellitus (T2DM) which is characterized by insulin resistance, caused by impairment of the pancreatic β-cell [280]. However, oxidative stress is believed to be the main cause of the onset and development of T2DM [281,282]. So, generation of ROS is a crucial factor in β-cell function [281]. Several studies have suggested that β-cells are highly susceptible to ROS because β-cells have lower antioxidant defenses, compared to other cells [281,283,284]. In addition, upon binding of its receptor, insulin commences a signaling cascade that elicits a mild oxidative burst of H_2_O_2_, which acts as a secondary messenger [285,286]. Many model studies have indicated that various antioxidant enzymes like selenoproteins are overexpressed in β-cells [281,285,287,288]. Indeed, high levels of GPx1 protect β-cells from H_2_O_2_, thus inhibiting insulin resistance in mice and human [287,288], but a deficiency of GPx1 raises insulin sensitivity in mice and human [289,290].

As oxidative stress is linked to the onset and progression of diabetes, antioxidant strategies would be a promising therapy for the treatment of diabetes. 

### 8.3. Viral Infections

Viral infections occur when the human body is invaded by viruses, such as human immunodeficiency virus (HIV) and severe acute respiratory syndrome—coronavirus 2 (SARSCoV2), that lead to many diseases. Viral infection often alters the intracellular redox homeostasis in the host cell by increasing ROS production, which enhances the viral replication [291,292,293,294]. Several selenoproteins, like glutathione peroxidases (GPxs) and thioredoxin reductase (TrxR), are important host antioxidants that may play an important role against viral infections by consuming ROS. 

#### 8.3.1. Human Immunodeficiency Virus (HIV)

HIV, a single-stranded RNA virus, belongs to the lentivirus family [295] that infects human immune cells, causing a weakened immune system [295,296]. A large amount of experimental evidence has suggested that HIV infection triggers significant oxidative stress in host cells [297]. During virus entry into host cells, the glycoprotein-120 (gp120) of HIV interacts with cell surface receptor CD4 [298]. The conformational change of gp120 occurs due to the reduction of disulfide bonds to dithiol in gp120 [299,300,301], enabling cell fusion and resulting in HIV entry into the host cell [297]. Moreover, the dithiol/disulfide exchange form of CD4 is also a key factor for the interaction of CD4 and gp120 [302,303,304,305]. Therefore, the redox status in CD4 and gp120 is essential for HIV entry into the host cell, suggesting that the inhibition of thiol/disulfide exchange may be a promising target for the treatment of HIV [300,301,304,305,306,307]. 

After viral entry into host cell, HIV attempts replication, where Tat, a HIV-encoded trans-activating protein [308], is required. The primary structure of Tat contains 101 amino acids and its active site is located in the Cys-rich region (amino acids 20–39) [309,310]. However, the activity of Tat is markedly inhibited by the reducing agent, suggesting that the intramolecular disulfide bonds of Tat are crucial for Tat function [311]. Overall, during viral infection (entry and replication), both gp120 and Tat alter the host redox status, which is compensated by several host-detoxifying enzymes like glutathione, glutathione peroxidase, thioredoxin, and thioredoxin reductase [306,307]. These detoxifying enzymes are able to transfer electrons to gp120 and Tat, thus regulating the dithiol/disulfide exchange in structural conformations. Indeed, both gp120 and Tat suppress GSH levels, leading to an increase in the GSSG/GSH ratio [312,313,314,315]. GSSG/GSH supplies electrons to GPxs and TrxR, suggesting that HIV-1 infection changes the expression of selenoproteins [316]. 

As GPx and TrxR are selenoproteins, they are influenced by selenium levels in the cell. Several studies show that selenium supplementation suppresses the progression of HIV and improves CD4 counts [317]. 

Therefore, the inactivation of these enzymes might be a promising target for the treatment of HIV [300,301,304,305,307,318]. By inhibiting GPx or TrxR functions, the electrons supply to GSH or Trx1 might be frozen, thereby settling the reduction of disulfide bonds to dithiol in gp120 and Tat, which is crucial for HIV entry and replication [319]. Indeed, auranofin is a well-known TrxR1 inhibitor that can inhibit HIV infection by inhibiting the reduction of disulfide bonds in gp120 [320]. 

#### 8.3.2. Coronavirus Disease-2019 (COVID-19)

The spread of Coronavirus Disease-2019 (COVID-19) caused a worldwide pandemic which has infected millions of people around the globe since 2019, caused by severe acute respiratory syndrome coronavirus-2 (SARS-CoV-2) [321,322,323]. The severity and mortality of COVID-19 are associated with various factors, including oxidative stress. The impairment of antioxidant defense is due to SARS-CoV-2 infection. Selenium and selenoproteins play a major role in combating oxidative stress in response to SARS-CoV-2 infections [324,325]. Several experiments from different countries have demonstrated that low serum levels are present in COVID-19 patients [326,327]. Interestingly, Se deficiency is also linked with the severity and mortality of other viral infections because deficiency of selenium reduces the activity of antioxidant enzymes leading to the amplification of ROS that induce viral replication [218,325,328,329,330,331]. However, currently, limited data on Se status in COVID-19 are available, and therefore further research is required to understand the role of Se in COVID-19. 

The first step of SARS-CoV-2 entry into the host cell involves the binding of viral spike proteins onto a surface receptor enzyme, angiotensin-converting enzyme 2 (ACE 2) [332,333]. Both ACE2 and viral spike proteins have many cysteine residues that are responsible for the conformational modulation of viral spike proteins through thiol–disulfide exchange, enabling virus entry into host cells and the consequent depletion of intracellular redox homeostasis [334,335,336]. This thiol–disulfide equilibrium in the extracellular surface region is maintained by several host antioxidant enzymes including GSH and Trx [334,335,336]. 

Low levels of GSH enhance cellular oxidative stress associated with uncontrolled SARS-CoV-2 infection and with down-regulation of TrxR and GPx4 [337,338,339]. The homeostasis of GSH/GSSG (thiol-disulfide) depends on TrxR and GPx, seleno enzymes which catalyze the thiol–disulfide reaction, facilitating the reduction of the disulfide bonds of viral spike proteins and ACE2, thus resulting in impairment of virus-receptor adducts [335], but no experimental data are available. 

In the process of virus replication, the main protease (Mpro), a highly conserved cysteine protease, cleaves polyproteins/peptides at multiple sites to produce multiple enzymatically active products [340,341]. Interestingly, the sequence of nsp13/14 junction (NVATLQ/A) of the Mpro cleavage site is similar to the GPx1 catalytic site sequence (NVASLU/G), wherein U (selenocysteine) lines up with Q (glutamine) in the Mpro sequence [342]. The U amino acid is not similar to the Q amino acid, but they are midrange in size and are polar amino acids in nature. The other two mismatched amino acid residues are S (serine) vs. T (threonine) and G (glycine) vs. A (alanine), both vary slightly by the presence of a methyl group [342,343]. Interestingly, GP_X_1 significantly binds with the inactive Mpro mutant (C145A), but no interaction is observed between GPx1 and wild-type Mpro [342,343]. Based on this, Gallardo et al investigated experimentally the cleavage of the GPx1 10-mer peptide by Mpro, but no cleavage was observed [324]. It can be concluded that selenocysteine is significantly different from glutamine at the cleavage site. So, GPx1 can be considered at least as a potential Mpro substrate [344]. Gallardo et al. have also shown experimentally that Mpro can target the TrxR. The predicted cleavage was observed when the Sec498Ser mutant TrxR was incubated in Mpro, killing the *C*-terminal redox center of TrxR [324]. It is obvious that TrxR and GPx are disordered, resulting in increasing oxidative stress, which is associated with the severity and mortality of COVID-19. 

### 8.4. Gestational Disorders

Selenium also plays an essential role in gestational health or during pregnancy, being one of the most important phases of a woman’s life and human reproduction [345]. It is reported that during pregnancy, the mother and fetus both demand more oxygen, resulting in the formation of more ROS, which is associated with miscarriage, premature rupture of membranes, preeclampsia, and intrauterine growth restriction [346,347]. Se performs its antioxidant activity by including Se as selenocysteine in the active sites of selenoproteins such as GPx and TrxR. Many studies have reported that Se deficiency enables poor levels of GPx and TrxR expression, leading to gestational disorders [345,348,349]. Therefore, supplementation of Se during pregnancy can reduce oxidative stress, resulting in a decrease in pregnancy complications [350,351]. It has been suggested that selenoproteins play a key role in modulating the production of ROS during pregnancy, fostering maternal and fetal diseases; however, more experimental studies are needed to elucidate the gestational disorders in detail.

### 8.5. Overview

**Figure 15:**
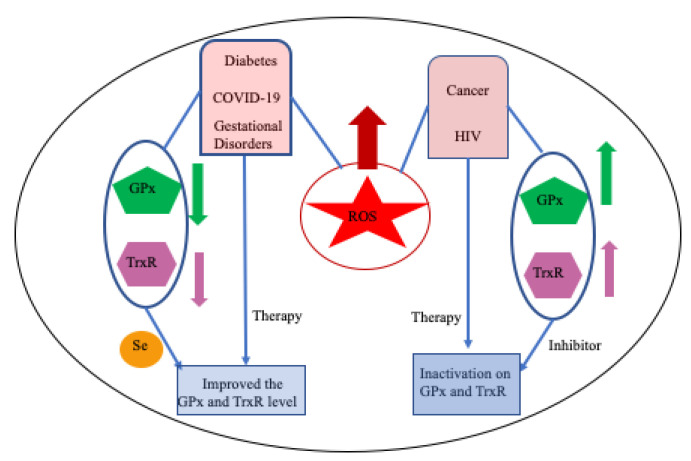
The cartoon illustrates all the connections between selenoproteins and various diseases. Arrows indicate up- and down-regulation.

Overall, SePs are involved in human health and diseases such as diabetes, cancer, viral infections, and gestational disorders [8,216,217,218,345]. These diseases mainly enhance the production of harmful ROS that modulate redox homeostasis in cells. Cell-containing SePs, particularly GPxs and TrxRs, are the key enzymes for maintaining redox homeostasis, which is the main contributing factor in the development and progression of various disease states [216,345]. Figure 15 presents the connection between the production of ROS (during disease states) and the expression of SePs (GPxs and TrxRs). Numerous therapies have suggested that the expression level of GPxs and TrxRs can be modulated, which may halt the diseases. It is suggested that SePs play a key role in maintaining the redox balance during various disease states; however, more experimental studies are needed to elucidate the detailed mechanisms of these diseases. 

## 9. Wrap-Up

Through the selenocysteine-containing enzymes above described, the following selected mechanistic and physiological roles of selenium were herein highlighted:

* Catalytic role in redox enzymes

The presence of one selenocysteine residue in an enzyme active site certainly introduces chemical features in the reaction mechanism which are not achievable with a “normal” cysteine. The selenocysteine selenol’s lower p*K*_a_ value (5.2, compared to 8.3 of cysteine thiol) favors its deprotonation and nucleophilic character at a physiological pH (exploited, for example, in Hases). Selenium’s preference for lower oxidation states and higher reactivity (compared to sulfur), as well as its ability to be easily regenerated (reduced back) from selenenic (RSeO^−^) and seleninic (R-SeOO^−^) forms and to participate in bridges with terminal sulfur atoms, represent other distinctive features (for example, to control cellular redox status and attain antioxidant activity). However, and remarkably, there are also striking examples, as is the case of FDHs, where replacing the selenium (selenocysteine) with sulfur (cysteine) does not affect at all the chemistry or the kinetics of the reaction.

* Physiological role in humans

Selenium is necessary for the conversion of the thyroid hormone thyroxine (T4) into its active form, triiodothyronine (T3), which is essential for regulating metabolism, growth, and development. Its role in immune system function is also key to enhancing the body’s defence mechanisms against infections and other immune-related conditions. In addition, selenoproteins are involved in DNA synthesis and repair processes, contributing to the maintenance of genetic stability and prevention of mutations, as well as in sperm motility and function and in preventing complications during pregnancy. Some studies suggest that selenium may have a role in reducing the risk of certain cancers, such as prostate, lung, and colorectal cancer. However, more research is needed to confirm these potential benefits.

## Figures and Tables

**Table 1:** Key features of some representative FDHs.

Active Site ^a^	Subunit Composition	Examples	Notes
**no metal**	α_2_ no redox-active cofactors	*Candida boidinii* FDH	● NAD-dependent
**W** **SeCys**	α W, [4Fe-4S]	*Clostridium carboxidivorans* FDH	● cytoplasmic? ● NAD-dependent
		*Thermoanaerobacter kivui* FDH	● hydrogen-dependent CO_2_ reductase
	αβ α: W, [4Fe-4S] β: 3 [4Fe-4S]	*Desulfovibrio gigas*, *Desulfovibrio alaskensis*, *Desulfovibrio vulgaris* FDHs	● periplasmic
	(αβ)_2_ α: W, [4Fe-4S] β: 3 [4Fe-4S]	*Moorella thermoacetica* FDH	● cytoplasmic ● NADP-dependent
	(αβγ)_2_ W, Fe	*Synthrobacter fumaroxidans* FDH	● periplasmic?
**W** **Cys**	αβ α: W, ≥ 1 Fe/S β: [4Fe-4S], FMN	*Methylobacterium extorquens* FDH	● cytoplasmic ● NAD-dependent
**Mo** **SeCys**	α Mo, [4Fe-4S]	*Escherichia coli* FDH H	● cytoplasmic ● formate–hydrogen lyase system
		*Acetobacterium woodii* FDH	● hydrogen-dependent CO_2_ reductase
	αβγ α: Mo, [4Fe-4S] β: 3 [4Fe-4S] γ: 4 *c* haems	*Desulfovibrio desulfuricans* FDH	● periplasmic
		*Desulfovibrio vulgaris* FDH	
	(αβγ)_3_ α: Mo, [4Fe-4S] β: 4 [4Fe-4S] γ: 2 *b* haems	*E. coli* FDH N	● membrane-bound periplasm-faced ● partner anaerobic nitrate–formate respir. system
		*E. coli* FDH O	● membrane-bound periplasm-faced ● partner microaerobic nitrate–formate respir. syst.
**Mo** **Cys**	α Mo, [4Fe-4S]	*Pectobacterium atrosepticum*, *Corynebacterium glutamicum* FDHs	● cytoplasmic
	αβ Mo, several Fe/S	*Clostridium pasteurianum* FDH	● cytoplasmic
	αβ Mo, FAD, several Fe/S, Zn	*Methanobacterium formicicum* FDH	● cytoplasmic ● F_420_-dependent
	αβγ α: Mo, [4Fe-4S] β: 4 [4Fe-4S] γ: 4 *b* haems	*Wolinella succinogenes* FDH	● membrane-bound
	(αβγ)_2_ α: Mo, [2Fe-2S], 4 [4Fe-4S] β: [4Fe-4S], FMN γ: [2Fe-2S]	*Cupriavidus necator*, *Rhodobacter capsulatus*, *Methylosinus trichosporium*, *Pseudomonas oxalatus* FDHs	● cytoplasmic ● NAD-dependent
	(αβγδ)_2_ Mo, ≥1 [2Fe–2S], ≥1 [4Fe–4S], FMN	*Methylosinus trichosporium* FDH	● cytoplasmic ● NAD-dependent
	(αβγδεω)_4_ α: 2 Zn β: Mo, [4Fe-4S] γ: 2 [4Fe-4S] γ: 4 *b* haems δ ε: 8 [4Fe-4S] ω	*Methanothermobacter wolfeiir* FMFDH	● cytoplasmic

^a^ Metal (molybdenum or tungsten) and residue (selenocysteine or cysteine) present in the active site of metal-dependent FDHs and FMFDHs.

**Table 2:** Comparison of the EPR properties of native and H_2_-reduced states of [NiFe]- and [NiFeSe]-Hases [140].

Hase	SRB	Localization	EPR g-Values Native	EPR g-Values H_2_ Red—Ni-C
**[NiFe]**	*D. gigas*	periplasm	2.31 2.23 2.02	2.19 2.14 2.02
**[NiFe]**	*D. desulfuricans* (ATCC 2774)	periplasm	2.32 2.16 2.02	2.19 2.14 2.02
**[NiFeSe]**	*D. desulfuricans* (Norway 4)	soluble	weak Ni(III) signals	2.20 2.15~2.0
**[NiFeSe]**	*D. salexigens*	periplasm	EPR silent	2.22 2.14 2.01
**[NiFeSe]**	*D. africanus*	soluble	EPR silent	2.21 2.17 2.01
**[NiFeSe]**	*D. baculatus*	soluble	weak Ni(III) signals	2.20 2.16 2.01

## Data Availability

The data presented in this study are available in article.

## Abbreviations

ACE-2: angiotensin-converting enzyme 2; COVID-19, Coronavirus Disease-2019; Cys-FDH, cysteine-containing FDH; Cys-FMFDH, cysteine-containing FMFDH; Cys-Mo-FDH, cysteine and molybdenum-containing FDH; Cys-W-FDH, cysteine and tungsten-containing FDH; Dios, iodothyronine deiodinases; DM, diabetes mellitus; EPR, electron paramagnetic resonance spectroscopy; FDH, formate dehydrogenase; [Fe]-Hase, [Fe]-hydrogenase; [FeFe]-Hase, [FeFe]-hydrogenases; FMFDH, *N*-formyl-methanofuran dehydrogenases; GPx, glutathione peroxidase; GSH, glutathione; Hase, hydrogenases; HIV, human immunodeficiency virus; IRD, inner-ring deiodination; Mpro, main proteases; [NiFe]-Hase, [NiFe]-hydrogenase; [NiSeFe]-Hase, [NiSeFe]-hydrogenase; ORD, outer-ring deiodination; ROS, reactive oxygen species; R-SeOH, selenenic acid; R-SeOOH, seleninic acid; SARS-CoV-2, severe acute respiratory syndrome coronavirus 2; SeCys-FDH, selenocysteine-containing FDH; SeCys-FMFDH, selebocysteine-containing FMFDH; SeCys-Mo-FDH, selenocysteine and molybdenum-containing FDH; SeCys-W-FDH, selenocysteine and tungsten-containing FDH; SePs, selenoproteins; T2DM, Type 2 diabetes mellitus; TGR, thioredoxin glutathione reductase; Trx, thioredoxin; TrxR, thioredoxin reductase; XAS, X-ray absorption spectroscopy.
